# Normal foot loading parameters and repeatability of the Footscan® platform system

**DOI:** 10.1186/s13047-017-0209-2

**Published:** 2017-07-17

**Authors:** Chao Xu, Xin-Xin Wen, Lu-Yu Huang, Lei Shang, Xi-Xia Cheng, Ya-Bo Yan, Wei Lei

**Affiliations:** 1 Department of Orthopedics, Xijing hospital, Fourth Military Medical University, No.15 Changle West Road, Xi’an, Shaanxi 710032 China; 2 Department of Orthopedics, No. 463 Hospital of Chinese PLA, Shenyang, 110042 China; 3 Department of Health Statistics, Faculty of Preventive Medicine, Fourth Military Medical University, Xi’an, 710032 China

**Keywords:** Footscan®, Plantar pressure, Repeatability, Intraclass correlation coefficients, Coefficients of variation, Normal

## Abstract

**Background:**

The Footscan® platform system is one of the most commonly used clinical tools for the measurement of the foot pressure. The present study was designed to assess the repeatability of the system and identify the range of loading parameters observed in the normal foot.

**Methods:**

Measurements were collected from 32 healthy participants, 15 females and 17 males, twice at an interval of 1 week. Peak pressure (PP), contact time (CT), contact area (CA), pressure-time integral (PTI), and maximum force (MaF) were recorded; these parameters were investigated in 10 areas of the foot: medial heel, lateral heel, midfoot, first to fifth metatarsals, hallux, and toes 2–5. The intra-session repeatability was evaluated by calculating the intraclass correlation coefficients (ICCs) and coefficients of variation (CVs) across the three repeated trials within the same session. The inter-session repeatability was assessed using the average of the three trials in each session to determine the ICCs and CVs.

**Results:**

The ICCs showed moderate to good repeatability for every variable of interest, and the CVs were all <28%. The highest zones of PP were found under the second and third metatarsals, followed by the medial heel. The CT was 68.5–82.8% of the total stance time under the metatarsal heads. CA was highest under the midfoot, PTI was highest under the second metatarsal, and MaF was highest under the medial heel.

**Conclusions:**

Footscan® platform system was found to be repeatable. Thus, it can be used as a valuable tool in the assessment of plantar pressure distribution, and the normal values of the foot loading parameters identified in this study can be employed to provide a reference range for the gait analysis performed by the Footscan® system.

## Background

Foot pressure measurement systems for quantitative gait analysis have become increasingly popular in research and clinical practice [1]. Such measuring systems can be used for distinguishing between normal and pathological gait [2], designing foot orthoses and in-shoe wedges [3, 4], classifying the foot types [5], and assessing the success of corrective foot surgery [6]. An ideal plantar pressure system should have the advantages of convenient use, comfort, economy, sanitation, safety, small occupation area, easy disassembly and transportation, highly accurate measurement, and satisfactory repeatability [7]. Since an increasing number of clinical decisions and treatment strategies are made based on the data collected by the plantar pressure systems, the knowledge about repeatability and normal reference values of the devices is critical before usage.

Presently, there are several brands of pressure measuring systems used in the clinic, including the in-shoe measurement systems (Novel Pedar®, TekScan F-Scan®, RS-Scan Insole®, WalkinSense®, and IBV Biofoot®) and platform systems (Novel Emed®, TekScan MatScan®, Medicapteurs S-Plate®, and the RS-Scan Footscan®) [8]. Most of these have proved to be reliable tools for quantifying the dynamic plantar pressure [7–16]. Some researchers utilized the “intraclass correlation coefficients (ICCs)” and/or “coefficients of variation (CVs)” as the evaluation criteria and found that the WalkinSense®, MatScan®, Pedar®, and Emed® systems demonstrated the acceptable reliability and repeatability, respectively [1, 8–10, 15]. Maetzler et al. [7] and Putti et al. [11, 14] took the “coefficient of repeatability” as the evaluation index and certified the repeatability of the Pedar® and Emed® systems, respectively. Although some clinical studies have been reported based on the Footscan® platform system, hitherto, only little information is available on the performance characteristics of this system. In 2010, Low et al. [17] reported an excellent reliability of the Footscan® pressure insoles. In addition, de Cock et al. [18] investigated the temporal characteristics of foot roll-over during jogging with the Footscan® platform and reported an adequate reliability while measuring the temporal parameter.

However, to the best of our knowledge, neither any of the previous publications have addressed the repeatability of the Footscan® platform system comprehensively, nor the ranges of the normal plantar pressure values have been identified for the healthy foot during level walking using this system. Thus, the present study was designed to assess the repeatability of the Footscan® platform system and establish a reference range for foot loading parameters, which can assist with the identification of pathological conditions.

## Methods

### Participants

Thirty-two healthy participants (*n* = 32), who were capable of ambulating independently, were recruited for assessment from the Fourth Military Medical University (Xi’an, Shaanxi Province, China). The participants were excluded if they suffered from foot pain within the previous 6 months, had any previous foot and ankle surgery, limb length discrepancies, foot deformities, or any clinical issues that could potentially affect their gait. The anthropometric data with respect to gender, age, body mass, height, and body mass index (BMI) were recorded for each participant prior to data collection. The study was approved by the Ethical Committee of the Fourth Military Medical University. Written informed consent was obtained from all participants before the commencement of the study.

### Experimental apparatus and set-up

The dynamic plantar pressure parameters were recorded using a Footscan® pressure plate (RSscan International, Olen, Belgium, 2096 × 472 × 18 mm, with 16,384 resistive sensors arranged in a 256 × 64 matrix at a resolution of 2 sensors/cm^2^, data acquisition frequency: 125 Hz, and pressure range: 0–200 N/cm^2^), which was connected to a computer. The platform was located at the center of a carpet with the same external dimension to provide a “complete platform” that was 4 m in length. According to the manufacturer’s manual, the Footscan® system was calibrated before each individuals test sessions. During calibration, the body weight and foot size of the participant were entered into the computer, and then, the participant was asked to walk on the plate. Subsequently, the analysis software would determine a recalibration factor for future measurements for the participant.

### Procedure

Testing sessions were conducted on two independent occasions with a 7-day interval [7–9, 19]. Both testing sessions were performed at approximately the same time of the day for each participant. A 7-day interval between the sessions was selected to ensure that the participants’ gait characteristics remained reasonably consistent [8]. In each testing session, three representative and reliable trials were recorded [1, 20]. All the measurements were recorded by the same observer (CX). A trial was considered as reliable when the following criteria were met: (1) at least one complete footprint for each foot, (2) a heel-strike pattern, (3) no obvious adjustment in gait pattern to contact the plate, (4) and the total stance time was within 10% of the individual mean values [21]. In each reliable trial, about 3–4 consecutive steps were captured, and only the most representative step of each foot was used in the statistical analysis [1]. Thus, in each testing session, three steps with each foot were recorded on the same platform.

All the participants received clear instructions about the testing protocols. Moreover, they were also requested to wear casual loose fitting clothing that did not impede the lower limb motion. Before collecting the dynamic data, all the participants completed 10 min acclimatization walking trails along the measuring platform. To prevent targeting, the participants were instructed not to look down at the platform while walking, rather look straight ahead at a fixed position away from the platform [20]. Based on the individual stride and step length obtained during the acclimatization trials, each participant determined a suitable starting position to ensure that three successive steps were taken prior to platform contact [22]. This approach ensured that the data were collected during mid-gait, which could minimize the effect of acceleration and deceleration at the start and end of each walk [23]. Subsequently, the participants underwent the pedobarographic tests barefoot at their comfortable walking pace. To prevent fatigue, each participant was required to take a 3 min recovery period between each trial [6]. The trial order was randomized between the participants. The same protocols were followed for both testing sessions.

### Data processing

**Fig. 1:**
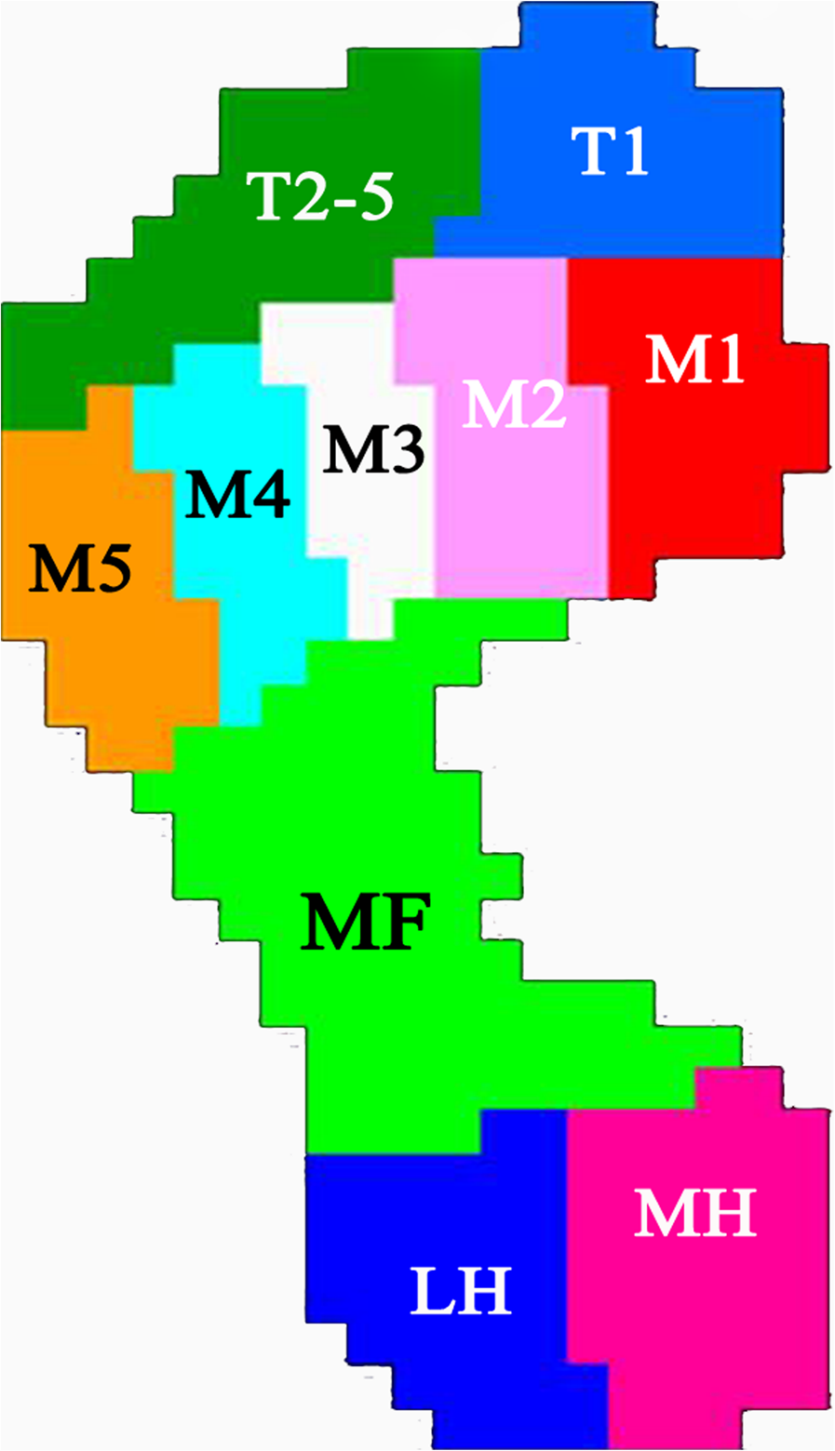
Schematic diagram for the 10 subdivided zones of the foot applied in the current study. Legend: The subdivided zones were T1) hallux, T2–5) toes 2–5, M1) first metatarsal, M2) second metatarsal, M3) third metatarsal, M4) fourth metatarsal, M5) fifth metatarsal, MF) midfoot, MH) medial heel, LH) lateral heel

The data were analyzed using Scientific Footscan® software (RSscan International), which automatically divided the foot into 10 masked zones: hallux (T1), toes 2–5 (T2–5), first to fifth metatarsals (M1, M2, M3, M4, and M5), midfoot (MF), medial heel (MH), and lateral heel (LH) (Fig. 1). After each measurement, a visual checking was made to assure that the anatomical structures fitted with the masked zones generated automatically. In the cases where the masked zones were unable to identify the foot, manual corrections were made to the applied mask, using a static image of the plantar surface of the participant’s foot as reference [24]. All the corrections were made by the same observer (CX). Five of the clinically most relevant variables were selected for evaluation: peak pressure (PP, kPa), contact time (CT, stance time%), contact area (CA, cm^2^), pressure-time integral (PTI, kPa s), and maximum force (MaF, N). In total, 50 parameters were assessed: 5 variables under 10 masked zones.

### Statistical analysis

Statistical analyses were performed using SPSS software (SPSS 19.0; SPSS Inc., Chicago, IL, USA). The data were explored for outliers and distribution. Normality was investigated using the one-sample Kolmogorov–Smirnov test. Normally distributed data were presented as mean (standard deviation, SD). In order to maintain the independence of the data, only the left foot of each participant was chosen for assessment [25–27]. The intra-session repeatability was evaluated via the calculation of the ICCs and CVs (expressed as a percentage of the mean) across the three repeated trials within the same session. The inter-session repeatability was assessed using the average of the three trials in each session to determine the ICCs and CVs. We considered ICC <0.50 as poor, 0.50–0.75 as moderate, and >0.75 as good [8]. The type of ICC used for this analysis was a one-way random ICC since the difference in the results between test sessions was random [28]. The 95% confidence intervals (CI) were also calculated with the ICCs.

Then, to assess the systematic differences between sessions, paired *t*-tests were used to compare the mean values of the foot loading parameters of interest for each masked zone. The significance level was set at 0.05. A reference range for foot loading parameters was calculated as “mean (six repeated trails on two sessions) ± 1.96 × SD” [11].

## Results

### Participant characteristics

The mean (SD, range) age, body mass, height, and BMI of the participants were 26.4 (5.0, range 19–36) years, 69.6 (11.3, range 49.5–100.0) kg, 174.1 (6.9, range 159–185) cm, and 22.9 (3.1, range 18.7–31.6) kg/m^2^, respectively. Of the 32 participants, 15 (46.9%) were females and 17 (53.1%) were males.

### Intra-session repeatability

ICCs and CVs across the three repeated trials within the same session ranged from 0.72 (MF)–0.91 (M2) and 12.4 (MH)–25.2% (MF) for PP, respectively, 0.65 (T2–5)–0.86 (LH) and 2.4 (M3)–19.7% (T2–5) for CT, respectively, 0.85 (T1 and T2–5)–0.95 (MF and MH) and 3.5 (MH)–10.4% (T2–5) for CA, respectively, 0.63 (T1)–0.83 (M2 and M3) and 12.0 (M3)–27.7% (T2–5) for PTI, respectively, and 0.78 (M5)–0.94 (T1) and 9.2 (MH)–25.2% (MF) for MaF, respectively. The average ICCs and CVs values for all regions of the foot were respectively, 0.81 and 17.1% for PP, 0.78 and 7.8% for CT, 0.89 and 6.7% for CA, 0.76 and 17.7% for PTI, and 0.84 and 17.1% for MaF. The regional intra-session ICCs for the PP were moderate in the MF zone and good in 9/10 masked zones. For the CT, the intra-session ICCs were moderate in T1 and T2–5 zones and good in the remaining 8 zones. For the CA, all the regional intra-session ICCs were good. For the PTI, the intra-session ICCs were moderate in T1, T2–5, M1, and MF zones and good in the remaining 6 zones. For the MaF, all the regional intra-session ICCs were good (Table 1).

**Table 1:** Regional intra-session ICCs and CVs for plantar loading measures

Zone	PP		CT		CA		PTI		MaF	
	ICCs (95% CI)	CVs (%)	ICCs (95% CI)	CVs (%)	ICCs (95% CI)	CVs (%)	ICCs (95% CI)	CVs (%)	ICCs (95% CI)	CVs (%)
T1	0.84 (0.75 - 0.90)	19.8	0.72 (0.58 - 0.82)	16.3	0.85 (0.77 - 0.91)	7.8	0.63 (0.44 - 0.76)	25.1	0.94 (0.90 - 0.96)	17.7
T2–5	0.76 (0.63 - 0.84)	22.0	0.65 (0.47 - 0.77)	19.7	0.85 (0.77 - 0.90)	10.4	0.70 (0.54 - 0.81)	27.7	0.89 (0.83 - 0.94)	23.0
M1	0.77 (0.65 - 0.85)	19.8	0.82 (0.73 - 0.88)	10.3	0.89 (0.83 - 0.93)	9.1	0.75 (0.62 - 0.84)	16.6	0.80 (0.68 - 0.88)	19.7
M2	0.91 (0.87 - 0.95)	13.5	0.79 (0.69 - 0.87)	2.9	0.86 (0.79 - 0.91)	6.2	0.83 (0.73 - 0.89)	13.0	0.88 (0.80 - 0.93)	14.8
M3	0.86 (0.79 - 0.91)	15.0	0.78 (0.67 - 0.86)	2.4	0.86 (0.79 - 0.91)	6.5	0.83 (0.74 - 0.89)	12.0	0.80 (0.68 - 0.88)	15.6
M4	0.80 (0.70 - 0.87)	14.0	0.79 (0.68 - 0.86)	2.6	0.87 (0.81 - 0.92)	5.7	0.77 (0.65 - 0.85)	14.3	0.79 (0.66 - 0.87)	17.2
M5	0.78 (0.67 - 0.86)	16.1	0.78 (0.66 - 0.85)	3.7	0.88 (0.82 - 0.93)	6.8	0.76 (0.63 - 0.85)	15.3	0.78 (0.64 - 0.87)	19.1
MF	0.72 (0.57 - 0.82)	25.2	0.81 (0.72 - 0.88)	7.7	0.95 (0.92 - 0.97)	7.4	0.72 (0.58 - 0.82)	22.8	0.89 (0.83 - 0.94)	25.2
MH	0.82 (0.72 - 0.88)	12.4	0.83 (0.75 - 0.89)	5.9	0.95 (0.92 - 0.97)	3.5	0.81 (0.71 - 0.88)	15.1	0.81 (0.70 - 0.89)	9.2
LH	0.81 (0.72 - 0.88)	13.1	0.86 (0.79 - 0.91)	6.0	0.94 (0.91 - 0.96)	3.8	0.81 (0.72 - 0.88)	15.4	0.80 (0.68 - 0.88)	9.3

*ICCs* intraclass correlation coefficients, *CVs* coefficient of variations, *PP* peak pressure, *CT* contact time, *CA* contact area, *PTI* pressure-time integral, *MaF* maximum force, *CI* confidence intervals, *T1* hallux, *T2–5* toes 2–5, *M1* first metatarsal, *M2* second metatarsal, *M3* third metatarsal, *M4* fourth metatarsal, *M5* fifth metatarsal, *MF* midfoot, *MH* medial heel, *LH* lateral heel

### Inter-session repeatability

The ICCs and CVs between the sessions ranged respectively, from 0.76 (MF)–0.92 (M3) and 7.8 (LH)–16.8% (T2–5) for PP, 0.81 (M5)–0.92 (MF) and 1.3 (M3 and M4)–9.1% (T2–5) for CT, 0.80 (M3)–0.97 (MF) and 2.4 (MH)–8.4% (T2–5) for CA, 0.75 (T1)–0.90 (LH) and 9.2 (M2)–23.0% (T2–5) for PTI, and 0.78 (M5)–0.93 (T1) and 7.7 (MH)–17.9% (MF) for MaF. The average ICCs and CVs values for all regions of the foot were respectively, 0.84 and 11.5% for PP, 0.87 and 4.5% for CT, 0.89 and 4.5% for CA, 0.81 and 14.2% for PTI, and 0.87 and 13.0% for MaF. All the regional inter-session ICCs for the PP, CT, CA, PTI, and MaF were good in the 10 masked zones (Table 2).

**Table 2:** Regional inter-session ICCs and CVs for plantar loading measures

Zone	PP		CT		CA		PTI		MaF	
	ICCs (95% CI)	CVs (%)	ICCs (95% CI)	CVs (%)	ICCs (95% CI)	CVs (%)	ICCs (95% CI)	CVs (%)	ICCs (95% CI)	CVs (%)
T1	0.88 (0.76 - 0.94)	12.6	0.91 (0.82 - 0.96)	9.0	0.92 (0.84 - 0.96)	3.7	0.75 (0.50 - 0.88)	19.0	0.93 (0.84 - 0.97)	14.9
T2–5	0.84 (0.67 - 0.92)	16.8	0.87 (0.74 - 0.94)	9.1	0.82 (0.62 - 0.91)	8.4	0.78 (0.55 - 0.89)	23.0	0.90 (0.77 - 0.96)	16.3
M1	0.81 (0.60 - 0.91)	13.1	0.88 (0.75 - 0.94)	8.2	0.91 (0.81 - 0.96)	5.9	0.80 (0.59 - 0.90)	14.7	0.90 (0.76 - 0.96)	15.8
M2	0.87 (0.74 - 0.94)	8.9	0.89 (0.77 - 0.94)	1.8	0.87 (0.74 - 0.94)	4.0	0.86 (0.71 - 0.93)	9.2	0.89 (0.74 - 0.95)	9.5
M3	0.92 (0.83 - 0.96)	9.8	0.82 (0.63 - 0.91)	1.3	0.80 (0.60 - 0.90)	4.7	0.82 (0.63 - 0.91)	10.3	0.85 (0.66 - 0.94)	11.9
M4	0.82 (0.64 - 0.91)	10.6	0.84 (0.67 - 0.92)	1.3	0.88 (0.76 - 0.94)	3.9	0.80 (0.60 - 0.90)	12.5	0.80 (0.53 - 0.91)	12.2
M5	0.77 (0.54 - 0.89)	10.8	0.81 (0.60 - 0.91)	2.0	0.81 (0.62 - 0.91)	5.1	0.79 (0.57 - 0.90)	12.4	0.78 (0.50 - 0.91)	14.2
MF	0.76 (0.51 - 0.88)	16.2	0.92 (0.83 - 0.96)	4.1	0.97 (0.93 - 0.98)	3.8	0.82 (0.64 - 0.91)	18.3	0.89 (0.75 - 0.95)	17.9
MH	0.86 (0.71 - 0.93)	8.2	0.87 (0.73 - 0.94)	3.8	0.96 (0.92 - 0.98)	2.4	0.80 (0.59 - 0.90)	13.1	0.90 (0.77 - 0.96)	7.7
LH	0.89 (0.77 - 0.95)	7.8	0.87 (0.74 - 0.94)	3.9	0.95 (0.89 - 0.97)	2.7	0.90 (0.80 - 0.95)	9.3	0.81 (0.55 - 0.92)	9.1

*ICCs* intraclass correlation coefficients, *CVs* coefficient of variations, *PP* peak pressure, *CT* contact time, *CA* contact area, *PTI* pressure-time integral, *MaF* maximum force, *CI* confidence intervals, *T1* hallux, *T2–5* toes 2–5, *M1* first metatarsal, *M2* second metatarsal, *M3* third metatarsal, *M4* fourth metatarsal, *M5* fifth metatarsal, *MF* midfoot, *MH* medial heel, *LH* lateral heel

### Systematic differences in the values between sessions

There were no systematic differences in the mean values of the PP, CT, CA, PTI, and MaF between sessions (Table 3).

**Table 3:** Comparison of the PP, CT, CA, PTI, and MaF in the 10 masked zones between sessions

Zone	PP (kPa)			CT (stance time%)			CA (cm^2^)			PTI (kPa s)			MaF (N)		
	Session 1	Session 2	*P*	Session 1	Session 2	*P*	Session 1	Session 2	*P*	Session 1	Session 2	*P*	Session 1	Session 2	*P*
T1	165.5 ± 51.7	157.7 ± 46.1	0.201	57.6 ± 15.6	57.8 ± 15.4	0.894	15.5 ± 2.3	15.5 ± 2.5	0.829	46.1 ± 18.2	34.7 ± 14.2	0.146	149.6 ± 54.2	142.3 ± 65.6	0.524
T2–5	50.2 ± 25.1	44.0 ± 19.5	0.226	41.8 ± 11.8	41.8 ± 12.2	0.985	16.2 ± 3.0	15.8 ± 3.5	0.387	9.2 ± 4.5	7.8 ± 3.4	0.299	72.5 ± 35.5	66.1 ± 29.1	0.136
M1	166.4 ± 42.2	190.2 ± 34.5	0.184	68.2 ± 12.2	68.7 ± 13.4	0.783	13.4 ± 2.9	13.2 ± 2.4	0.414	42.9 ± 15.7	48.5 ± 16.3	0.645	153.8 ± 65.4	166.4 ± 69.1	0.286
M2	380.9 ± 91.8	354.0 ± 84.2	0.068	79.9 ± 4.6	79.9 ± 4.1	0.951	11.5 ± 1.7	11.7 ± 1.3	0.345	90.8 ± 26.4	86.0 ± 23.6	0.630	314.8 ± 74.7	312.2 ± 83.5	0.813
M3	349.3 ± 105.8	339.9 ± 97.0	0.154	82.9 ± 3.9	82.8 ± 3.6	0.854	13.8 ± 0.9	11.2 ± 1.4	0.254	87.7 ± 30.6	82.7 ± 25.6	0.482	375.5 ± 97.2	364.8 ± 89.9	0.421
M4	245.1 ± 61.7	224.0 ± 50.9	0.091	82.2 ± 3.5	82.3 ± 4.3	0.880	9.7 ± 0.9	9.9 ± 1.5	0.082	55.2 ± 21.9	54.2 ± 18.7	0.328	215.0 ± 61.4	209.4 ± 56.2	0.509
M5	119.0 ± 33.8	113.8 ± 28.6	0.465	77.9 ± 4.8	77.7 ± 5.2	0.804	12.9 ± 1.7	12.8 ± 2.2	0.849	33.0 ± 15.5	30.8 ± 13.6	0.617	155.0 ± 59.6	143.3 ± 52.4	0.115
MF	64.6 ± 30.2	65.8 ± 24.6	0.718	62.9 ± 10.0	63.0 ± 8.4	0.176	38.4 ± 7.6	38.4 ± 7.8	0.959	16.7 ± 8.1	14.9 ± 6.3	0.469	194.2 ± 101.8	200.1 ± 92.7	0.654
MH	253.0 ± 54.1	258.4 ± 46.2	0.229	59.2 ± 5.5	58.2 ± 7.4	0.180	21.9 ± 2.2	21.8 ± 2.7	0.490	57.5 ± 18.1	48.5 ± 14.9	0.303	457.0 ± 100.5	464.5 ± 112.9	0.275
LH	220.5 ± 48.2	219.5 ± 43.2	0.838	57.7 ± 6.0	58.1 ± 7.3	0.245	19.3 ± 2.1	19.3 ± 2.2	0.885	46.7 ± 15.9	45.5 ± 12.7	0.581	341.8 ± 85.7	333.3 ± 79.2	0.557

Values are expressed as means ± standard deviation

*PP* peak pressure, *CT* contact time, *CA* contact area, *PTI* pressure-time integral, *MaF* maximum force, *T1* hallux, *T2–5* toes 2–5, *M1* first metatarsal, *M2* second metatarsal, *M3* third metatarsal, *M4* fourth metatarsal, *M5* fifth metatarsal, *MF* midfoot, *MH* medial heel, *LH* lateral heel

### Normal foot loading parameters

The highest PP was recorded under the M2 zone [367.5 (87.9) kPa], followed by the M3 zone [344.6 (101.4) kPa], the MH zone [255.7 (50.1) kPa], and the M4 zone [234.6 (56.3) kPa]. The T2–5 [47.1 (22.3) kPa] and MF [65.3 (27.3) kPa] zones showed the lowest PP (Table 4). When CT was expressed as a percentage of the total stance time, the M3 zone [82.8 (3.7) %] was the longest in contact with the platform, closely followed by the M4 zone [82.2 (3.9) %], the M2 zone [79.9 (4.3) %], and the M5 zone [77.8 (5.0) %]. The T2–5 zone [41.8 (12.0) %] had the shortest CT (Table 4). In the case of CA, the MF zone [38.4 (7.7) cm^2^] made the largest contact with the Footscan® platform, followed by the MH zone [21.8 (2.4) cm^2^], the LH zone [19.3 (2.1) cm^2^], the T2–5 zone [16.0 (3.2) cm^2^], and the T1 zone [15.5 (2.4) cm^2^], successively; the CA of the M4 zone [9.8 (1.2) cm^2^] was the smallest (Table 4). The PTI was highest in the M2 zone [88.3 (25.0) kPa s], followed by the M3 zone [85.2 (28.1) kPa s] and the M4 zone [54.7 (20.3) kPa s]. The T2–5 zone [8.5 (3.9) kPa s] had the lowest PTI (Table 4). The highest MaF was recorded under the MH zone [460.7 (106.7) N], followed by the M3 zone [370.1 (93.5) N] and the LH zone [337.5 (82.4) N]. The T2–5 [69.3 (32.3) N] and T1 [146.0 (59.9) N] zones had the lowest MaF (Table 4). The reference ranges for PP, CT, CA, PTI, and MaF are presented in Table 4.

**Table 4:** Mean, SD, and reference range for the PP, CT, CA, PTI, and MaF for the 10 regions of the foot

Zone	PP (kPa)			CT (stance time%)			CA (cm^2^)			PTI (kPa s)			MaF (N)		
	Mean (SD)	Reference range		Mean (SD)	Reference range		Mean (SD)	Reference range		Mean (SD)	Reference range		Mean (SD)	Reference range	
		LB	UB		LB	UB		LB	UB		LB	UB		LB	UB
T1	161.6 (48.9)	65.8	257.4	57.7 (15.5)	27.3	88.1	15.5 (2.4)	10.8	20.2	40.4 (16.2)	8.6	72.2	146.0 (59.9)	28.6	263.4
T2–5	47.1 (22.3)	3.4	90.8	41.8 (12.0)	18.3	65.3	16.0 (3.2)	9.7	22.3	8.5 (3.9)	0.9	16.1	69.3 (32.3)	6.0	132.6
M1	178.3 (38.3)	103.2	253.4	68.5 (12.8)	43.4	93.6	13.3 (2.6)	8.2	18.4	45.7 (15.9)	14.5	76.9	160.1 (67.1)	28.6	291.6
M2	367.5 (87.9)	195.2	539.8	79.9 (4.3)	71.5	88.3	11.6 (1.5)	8.7	14.5	88.3 (25.0)	39.3	137.3	313.5 (78.9)	158.9	468.1
M3	344.6 (101.4)	145.9	543.3	82.8 (3.7)	75.5	90.1	12.5 (1.1)	10.3	14.7	85.2 (28.1)	30.1	140.3	370.1 (93.5)	186.8	553.4
M4	234.6 (56.3)	124.3	344.9	82.2 (3.9)	74.6	89.8	9.8 (1.2)	7.4	12.2	54.7 (20.3)	14.9	94.5	212.1 (58.7)	97.0	327.2
M5	116.4 (31.2)	55.2	177.6	77.8 (5.0)	68.0	87.6	12.8 (1.9)	9.1	16.5	31.9 (14.5)	3.5	60.3	149.1 (55.9)	39.5	258.7
MF	65.3 (27.3)	11.8	118.8	62.9 (9.2)	44.9	80.9	38.4 (7.7)	23.3	53.5	15.8 (7.1)	1.9	29.7	197.1 (97.2)	6.6	387.6
MH	255.7 (50.1)	157.5	353.9	58.7 (6.4)	46.2	71.2	21.8 (2.4)	17.1	26.5	53.0 (16.5)	20.7	85.3	460.7 (106.7)	251.6	669.8
LH	220.0 (45.7)	130.4	309.6	57.9 (6.6)	45.0	70.8	19.3 (2.1)	15.2	23.4	46.1 (14.2)	18.3	73.9	337.5 (82.4)	176.0	499.0

*PP* peak pressure, *CT* contact time, *CA* contact area, *PTI* pressure-time integral, *MaF* maximum force, *SD* standard deviation, *T1* hallux, *T2–5* toes 2–5, *M1* first metatarsal, *M2* second metatarsal, *M3* third metatarsal, *M4* fourth metatarsal, *M5* fifth metatarsal, *MF* midfoot, *MH* medial heel, *LH* lateral heel, *LB* lower bound, *UB* upper bound

## Discussion

Plantar pressure measurement is a clinical tool for assessing foot pathology, which has been regarded as an integral component while formulating the patient’s intervention plans [29]. The Footscan® platform system is commonly employed in the research and clinical setting, and therefore, it is essential to determine the repeatability of this system and identify the standard pressure values.

The mid-gait and two-step protocols are the commonly used methods to collect the foot pressure data [20, 30]. In some other plantar pressure system reliability studies, the mid-gait protocol was adopted [7, 14]. The researchers believed that the mid-gait was an optimal representative of the normal gait [14], and the participants were allowed extra time to acclimatize themselves to the mid-gait protocol to improve the quality of measurement [7]. Compared with the mid-gait protocol, the two-step protocol was simpler and time-saving [20, 31], and thus, it might be more suitable for patients with severe gait or coordination problems and those who experience difficulty in accomplishing a prolonged plantar pressure test [31]. However, some authors reported that the two-step protocol produced longer CT [20, 30, 32] than the mid-gait protocol. In addition, Wearing et al. [30] reported that the two-step protocol elicited reductions in both the PP and MaF beneath the heel. Hence, the two-step protocol might not resemble the natural gait [7]. In the present study, all the participants were healthy and capable of ambulating independently; thus, to record a natural gait, the mid-gait protocol was applied. Van der Leeden et al. [20] reported that a minimum of three measurements were sufficient for obtaining a consistent average. In the present study, three representative trials were recorded per testing session.

Herein, we assessed the repeatability of the Footscan® platform system for the 50 parameters of interest by calculating the ICCs and CVs. Considering the values of ICCs, every dynamic parameter analyzed showed moderate to good repeatability. For the intra-session repeatability, the majority (86%, 43/50) of the parameters had good repeatability (ICCs > 0.75), and the mean CV values for PP, CT, CA, PTI, and MaF were 17.1, 7.8, 6.7, 17.7, and 17.1%, respectively. For the inter-session repeatability, all the parameters showed good repeatability, and the mean CV values for PP, CT, CA, PTI, and MaF were 11.5, 4.5, 4.5, 14.2, and 13.0%, respectively. Several other studies demonstrated the intra- and inter-session repeatability of different pressure measuring systems, and the results were comparable to those observed in the present study with ICCs > 0.75 [17] and CVs < 20% [8, 33]. As is well-known, human gait is a rhythmical oscillation, and the foot steps are not identical in every gait cycle [34, 35]. Therefore, the level of ICCs and CVs achieved in the present study is clinically acceptable, which suggests that the Footscan® system is repeatable. In addition, we found that the inter-session repeatability was higher than the intra-session repeatability owing to the inter-session measurements being calculated with an average of three trials. Therefore, using a single trial to capture a participant’s foot loading parameters is not sufficient, and multiple trials should be averaged to decrease the variability of gait, as physiological fluctuations between trials are inevitable [23].

It is worth noting that the T2–5 and MF zones exhibited lower ICCs and higher CVs than the other zones in the variables that were analyzed. The findings were consistent with those from previous studies that used the Emed® and MatScan® platform systems [8, 15] and the Pedar® in-shoe system [9], which indicated the poorest repeatability in T2–5 and MF zones. The authors attributed the greater variability to the inherent variability in these regions during gait and relative smaller force and pressure exerted upon the T2–5 and MF zones [8, 15]. The present results supported the explanation. We found that areas with lower PP and MaF, such as the T2–5 and MF zones, showed lower repeatability than the more loaded regions, such as the M2, M3, MH, and LH zones. These findings are clinically important because the regions of the foot with high plantar pressures are good indicators of potential injury [36, 37]. Therefore, a higher repeatability in these zones is highly desirable for clinical applications [15]. In addition, the information elicited from the analysis of the plantar pressures and forces under the T2–5 and MF zones should be treated with caution.

In the present study, we also identified the ranges for PP under the healthy foot. Clinically, PP is the most relied upon plantar pressure parameter [7]. In the current investigation, the higher PP values were found under the M2, M3, and MH regions, and the lower ones were found under the T2–5 and MF zones. These findings were in agreement with the previous reports using other plantar pressure platform systems [14, 26, 38]. Some authors reported that the T1 zone exhibited the biggest PP [7], while we found the highest PP value under the M2 zone, closely followed by that of the M3 zone. Different observation results of the PP distributions could be attributed to different divisions of the foot, softwares used for analysis, participants, test protocols, experimental conditions, sensor characteristics, and measuring technologies [14, 38].

It’s important to note that the mean PP values obtained in this study using the Footscan® system are lower than that from the other studies [7, 14, 26]. In 1996, Davis et al. [39] recommended collecting the plantar pressure data with sensors that have dimensions ≤6.36 mm × 6.18 mm. Subsequently, Urry et al. [40, 41] used a pressure platform with a sensor size of 5 mm × 6 mm to determine the accuracy of the footprint CA measurements and the geometric indexes derived from the footprints. The studies reported that platforms with smaller sensor size and greater spatial resolution might produce a more accurate measurement of the footprint parameters [40, 41]. Another study by Urry et al. [42] recommended using platforms that have sensors of 5 mm × 5 mm or less. They believed that platforms with larger sensors would provide an underestimation of the peak pressure. According to the manufacturer’s manual, the sensor dimensions of the Footscan® platform were 7.62 mm × 5.08 mm. The relatively larger sensors and lower spatial resolution (2 sensors/cm^2^) of the Footscan® platform might affect the accuracy of the measurements. This reminds us that the Footscan® system might be more appropriate for the comparisons of conditions using the same system rather than situations where absolute values are required to determine the clinical condition of the participants [17].

Consistent with previous studies using other plantar pressure platform systems [7, 14, 38] and in-shoe system [11], CT was longest in the metatarsal regions, and the M3, M4, and M2 zones were the top 3 regions showing long CT. The metatarsal heads bore weight for 68.5–82.8% of the stance time, which is comparable with previous studies using the platform [7, 14] and in-shoe systems [9, 11].

CA is an important plantar pressure variable, and the combination of CA and PP can provide a lot of information for the prediction of potential damage. In the current study, CA was highest under the heel region (MH + LH zones), followed by the MF zone. Meanwhile, the smaller CA was recorded under the metatarsal regions which may lead to higher PP [14]. These results are consistent with previous studies using the platform [7, 14] and in-shoe systems [9, 11].

PTI of the whole stance phase reflects the integrated effects of pressure and time, which is related to foot pains [43, 44] and skin problems such as diabetic foot ulcers [45]. Monitoring the PTI may serve as a valuable strategy for the early prediction and prevention of the pathological conditions. The PTI values found in this study were higher under the M2 and M3 zones, and lower under the MF and T2–5 zones, supporting the findings of previous investigators who used the platform [7, 14] and in-shoe systems [9, 11].

MaF is a commonly used dynamic plantar pressure parameter. In the present study, the higher MaF values were found under the MH, M3, LH, and M2 zones, and the lower ones were found under the T2–5 and T1 zones. In addition, the T2–5 and MF zones exhibited higher intra- and inter-session CVs than the other zones for the MaF. These findings were in agreement with previous reports [8], which used the TekScan MatScan® platform system.

Nevertheless, there are some limitations of this study that should be pointed out. First, this study is limited by a small number of participants, which might reduce the reliability of the results. Second, all the participants in the present study were young healthy adults, and thus, our findings cannot necessarily be extrapolated to other clinical populations. Future investigations should focus on the repeatability of the plantar pressure measurement in patients with gait problems. Third, the selection of a representative step and manual corrections to the masked zones were subjective; the need for a standardized method is required. Finally, since different systems have different performance characteristics, the range of foot loading parameters identified in the current study cannot be considered when using other brands of systems.

## Conclusions

The results of this study indicate that the Footscan® system is a reliable instrument for assessing the dynamic plantar pressure distributions during barefoot level walking in healthy participants. The system displayed satisfactory repeatability for the selected parameters that are commonly used in clinical investigations. The ranges of PP, CT, CA, PTI, and MaF values have been analyzed and can be used to assist with the identification of cases with foot problems. Moreover, the ranges should be used cautiously, and the overall clinical situation must be taken into consideration when making clinical judgments and treatment recommendations.

## Abbreviations

BMI: body mass index
CA: contact area
CI: confidence intervals
CT: contact time
CVs: coefficients of variation
ICCs: intraclass correlation coefficients
LH: lateral heel
M1: first metatarsal
M2: second metatarsal
M3: third metatarsal
M4: fourth metatarsal
M5: fifth metatarsal
MF: midfoot
MaF: maximum force
MH: medial heel
PP: peak pressure
PTI: pressure-time integral
SD: standard deviation
T1: hallux
T2–5: toes 2–5
